# Altered Toll-Like Receptor Signalling in Children with Down Syndrome

**DOI:** 10.1155/2019/4068734

**Published:** 2019-09-12

**Authors:** Dean Huggard, W. J. Koay, Lynne Kelly, Fiona McGrane, Emer Ryan, Niamh Lagan, Edna Roche, Joanne Balfe, T. Ronan Leahy, Orla Franklin, Ana Moreno-Oliveira, Ashanty M. Melo, Derek G. Doherty, Eleanor J. Molloy

**Affiliations:** ^1^Paediatrics, Trinity College, The University of Dublin, Dublin, Ireland; ^2^Trinity Translational Medicine Institute (TTMI), Trinity College Dublin, Dublin, Ireland; ^3^Paediatrics, Tallaght Hospital, Dublin, Ireland; ^4^National Children's Research Centre, Our Lady's Children's Hospital, Crumlin, Dublin, Ireland; ^5^Immunology, Our Lady's Children's Hospital, Crumlin, Dublin, Ireland; ^6^Cardiology, Our Lady's Children's Hospital, Crumlin, Dublin, Ireland; ^7^Coombe Women and Infants University Hospital, Crumlin, Dublin, Ireland; ^8^Neonatology, Our Lady's Children's Hospital, Crumlin, Dublin, Ireland

## Abstract

Toll-like receptors (TLRs) are the key in initiating innate immune responses. TLR2 is crucial in recognising lipopeptides from gram-positive bacteria and is implicated in chronic inflammation. Children with Down syndrome (DS) are prone to infections from these pathogens and have an increased risk of autoimmunity. Sparstolonin B (SsnB) is a TLR antagonist which attenuates cytokine production and improves outcomes in sepsis. We hypothesised that TLR signalling may be abnormal in children with DS and contribute to their clinical phenotype. We evaluated TLR pathways in 3 ways: determining the expression of TLR2 on the surface of neutrophils and monocytes by flow cytometry, examining the gene expression of key regulatory proteins involved in TLR signal propagation, MyD88, IRAK4, and TRIF, by quantitative PCR, and lastly determining the cytokine production by ELISA following immunomodulation with proinflammatory stimuli (lipopolysaccharide (LPS), Pam3Csk4) and the anti-inflammatory agent SsnB. We report TLR2 expression being significantly increased on neutrophils, total monocytes, and intermediate and nonclassical monocytes in children with DS (*n* = 20, mean age 8.8 ± SD 5.3 years, female *n* = 11) compared to controls (*n* = 15, mean age 6.2 ± 4.2 years, female *n* = 5). At baseline, the expression of MyD88 was significantly lower, and TRIF significantly raised in children with DS. The TLR antagonist SsnB was effective in reducing TLR2 and CD11b expression and abrogating cytokine production in both cohorts. We conclude that TLR signalling and the TLR2 pathway are dysregulated in DS, and this disparate innate immunity may contribute to chronic inflammation in DS. SsnB attenuates proinflammatory mediators and may be of therapeutic benefit.

## 1. Introduction

Down syndrome (DS) is caused by extra genetic material from chromosome 21 and is the most common of the chromosomal abnormalities affecting 1 in 700 births in the USA [1]. It is associated with a myriad of health complications including developmental delay, congenital heart disease, gastrointestinal atresias, acute leukaemia, and obstructive sleep apnoea [2]. Immune dysregulation is another important feature of DS such as reduced T and B lymphocyte counts [3, 4], altered serum cytokines [5, 6], and suboptimal antibody responses to immunisation [7–9]. However, there are key aspects of the immune system that, to our knowledge, have not been examined in this cohort previously.

Toll-like receptors (TLRs) and their activation are crucial in initiating the innate immune response, while also linking the adaptive response to infection [10]. Activation of these TLRs initiates downstream signalling pathways and recruitment of a constellation of adaptor proteins: myeloid differentiation primary response gene 88 (MyD88), MyD88 adaptor-like protein (MAL), IL-1R-associated kinase (IRAK) family, and TIR-domain-containing adaptor protein-inducing interferon-*β* (TRIF), which in turn stimulate downstream kinases (JNK, ERK, and MAPKs), leading to nuclear translocation of an increase in the transcription factors, including nuclear factor kappa B (NF-*κ*B) and interferon regulatory factor-3 (IRF-3), ultimately leading to the production of proinflammatory cytokines [11]. Strict regulation of these TLR pathways is crucial in achieving protection from infection but also avoiding damage from excess cytokine production which can lead to worse outcomes, acutely in sepsis or in chronic inflammation resulting in autoimmunity, both of these sequelae occurring more frequently in DS [12, 13].

TLR2 is involved in detecting bacterial infection and in chronic inflammation and is located at the cell membrane, where it recognises and binds to signal molecules. These molecules are derived from microorganisms such as bacteria, viruses, or fungi exhibiting pathogen-associated molecular patterns (PAMPs, e.g., peptidoglycan, lipoproteins, lipoarabinomannan, lipoteichoic acid, zymosan, and glycolipids) or from dying endogenous cells bearing damage-associated molecular patterns (DAMPs, e.g., endogenous DAMPs, heat shock protein 70 (HSP70), Snapin, and hyaluronic acid) [14, 15]. Although lipopeptides from gram-positive bacteria and commercial TLR2 agonists such as Pam3Cys-Ser-(Lys)4-3HCL (Pam3Csk4, 16) are classically associated with activation of this receptor, there is evidence that lipopolysaccharide (LPS) endotoxin can also produce robust increases in TLR2 expression [16, 17]. Both *in vivo* and *in vitro* experiments have demonstrated significant increases in monocyte TLR2 after LPS treatment [16].

The significance of TLR2 and its affinity for constituents of gram-positive bacteria are of particular relevance in DS as individuals with DS are more prone to infection from this group of pathogens. Children with DS are at increased risk of infections and have greater risk of admission to a hospital with respiratory tract infections (RTIs), where they are more likely to have a protracted stay and require intensive care support [18]. Gram-positive bacteria such as *Streptococcus pneumoniae* and *Staphylococcus aureus* are the causative organisms in many of these cases, with the former also a significant contributor to recurrent otitis media with effusion (OME), which often leads to conductive hearing loss in these children [4]. Aggressive longstanding periodontitis resulting from persistent infection and chronic inflammation is seen regularly in this population [19]. Elevated TLR2 expression is found in the oral mucosa of patients with chronic periodontitis compared to controls [20, 21].

Dysregulated TLR2 signalling causes autoinflammation due to unchecked proinflammatory cytokine release. This can lead to several autoimmune and inflammatory conditions such as rheumatoid arthritis, systemic lupus erythematosus, atherosclerosis, and sepsis [22]. Children with DS are more susceptible to similar conditions like arthropathy and thyroid and coeliac disease [23] and have increased mortality from sepsis [13]. CD11b is a cell surface receptor which indicates activation and mediates neutrophil and monocyte diapedesis and adhesion [24]. Abnormal neutrophil migration and adherence have been linked to an increased risk of infection in neonates and adults [25]. Therefore, developing and utilising TLR antagonists or inhibitors of these pathways may prove to be of clinical benefit. Sparstolonin B (SsnB) is a natural isocoumarin compound which is derived from the roots of plant species such as *Sparganium stoloniferum* and *Scirpus yagara* and has been shown to reduce inflammation [26]. This compound acts as a selective TLR2 and TLR4 antagonist by preferentially limiting the association of MyD88 with TLR2 and TLR4 and reduces general NF-*κ*B activity [27]. SsnB has the potential to abrogate proinflammatory cytokine release, and macrophages treated with LPS or Pam3Csk4 have reduced expression of proinflammatory cytokines such as interleukin 1*β* (IL-1*β*), interleukin 6 (IL-6), and tumour necrosis factor alpha (TNF-*α*) [28].

We hypothesised that TLR signalling may be abnormal in children with DS and that altered TLR2 contributes to a clinical phenotype at increased risk of gram-positive infection, chronic inflammation, and autoimmunity. We aimed to evaluate TLR pathways in 3 ways: by determining the expression of TLR2 on the surface of neutrophils, monocytes, and their subsets, by examining the gene expression of key regulatory proteins involved in TLR signal propagation, MyD88, IRAK4, and TRIF, and lastly by determining the cytokine production at baseline and following immunomodulation with proinflammatory stimuli (LPS, Pam3Csk4) and the anti-inflammatory agent SsnB.

## 2. Materials and Methods

### 2.1. Study Population

This study was approved by the Ethics committees in the National Children's Hospital, Tallaght, and Our Lady's Children's Hospital, Crumlin (OLCHC), Dublin, Ireland. All families and participants received verbal and documented information on the study, and written consent was obtained prior to recruitment. There were two patient cohorts enrolled: (a) children with Down syndrome < 16 years old attending the dedicated Down syndrome clinic, or OLCHC, and (b) age-matched patients (controls) attending phlebotomy or day case procedures; in this instance, blood sampling occurred at the induction of general anaesthetic. Both groups had no recent evidence of infection or fever.

### 2.2. Patient Characteristics

There were 20 children with Down syndrome (DS) with a mean ± SD age of 8.79 ± 5.27 years (y) of which 11 were female and 15 controls with a mean age of 6.22 ± 4.22 y, of which 5 were female. In the DS cohort, children with a history of significant congenital heart disease requiring surgery in infancy (*n* = 8) were all clinically stable with no further cardiology intervention. Both groups were afebrile at the time of blood sampling with no recent history of infection.

### 2.3. Experimental Design

We utilised the same experimental methods (as outlined below), as per our previous paper [29], appraising TLR4 and CD11b expression on neutrophils and monocytes in DS at baseline and in response to LPS. Blood samples (1-3 mL) for *in vitro* experiments were collected in a sodium citrate anticoagulated blood tube and analysed within two hours of phlebotomy. Whole blood was incubated at 37°C for 1 hour with the proinflammatory stimulants lipopolysaccharide (LPS; *E. coli* 0111:B4, Sigma Life Science, Wicklow, Ireland) 10 ng/mL, Pam3Csk4 (Tocris Bio-Techne, Abingdon, UK) 5 ng/mL, and the TLR antagonist sparstolonin B (SsnB; Sigma Life Science, Wicklow, Ireland) 10 *μ*M and in combination.

### 2.4. Antibodies and Flow Cytometry

Blood samples were incubated with a dead cell stain (Fixable Viability Dye eFluor 506, Invitrogen, California, USA), diluted to working concentration in phosphate-buffered saline (PBS). The following fluorochrome-labelled monoclonal antibodies (mAb) were added to each sample: CD14-PerCP, CD15-PECy7, CD16-FITC, CD66b-Pacific Blue, and TLR2-APC (BioLegend®, California, USA) and PE-labelled CD11b (BD Biosciences, Oxford, UK). PBA buffer (PBS containing 1% bovine serum albumin and 0.02% sodium azide) was used to make up the antibody cocktail. Samples were incubated in the dark for 15 minutes. Next, 1 mL of FACS lysing solution (BD Biosciences, Oxford, UK) was added to each tube, and the samples were then incubated for 15 minutes in the dark. Cells were pelleted by centrifugation at 450g for 7 minutes at room temperature, washed twice with PBA buffer, and fixed in 300 *μ*L of 1% paraformaldehyde. The final cell pellet was resuspended in 100 *μ*L PBA buffer and analysed on a BD FACSCanto II flow cytometer.

The expression of TLR2 and CD11b antigens on the surface of neutrophils and monocytes was evaluated by flow cytometry. Neutrophils were delineated based on SSC-A and CD66b+ positivity as previously described [30], and monocytes were defined based on SSC-A and CD66b negativity and their subsets based on CD14 and CD16 expression: classical (CD14+/CD16-), intermediate (CD14+/CD16+), and nonclassical (CD14dim/CD16+). A minimum of 10,000 events were collated, and relative expression of TLR2 and CD11b was expressed as mean fluorescence intensity (MFI). Flow cytometry data was analysed using FlowJo software (Oregon, USA). Every sample was processed and analysed by the same researcher (DH) thereby reducing variability in results.

### 2.5. RNA Extraction, cDNA Synthesis, and RT-PCR

Following incubation of samples with proinflammatory stimulants, 1 mL of TRIzol (Thermo Fisher) solution was added to 0.3 mL of whole blood. The samples were incubated for 5 min at room temperature followed by the addition of chloroform. Following lysis, the aqueous phase was used to isolate RNA, as per the manufacturer's instructions (Invitrogen, USA). RNA purity and concentration were determined by using the NanoDrop ND-100 Spectrophotometer and analysed using ND-1000 ver.3.1.2 software. Total RNA, 1 *μ*g, was reverse transcribed to single-stranded cDNA using the High-Capacity cDNA Archive Kit (Applied Biosystems) following the manufacturer's protocol and stored at -80°C until use. The settings for amplification were 10 min at 25°C and 120 min at 37°C, and 5 sec at 85°C and then holding at 4°C. The evaluation of gene expression was performed by TaqMan® RT-PCR. Commercially available TaqMan® primer and probe combinations were used to detect the expression of the following genes, MyD88 (NM 001172567.1), TRIF (NM_182919.3), and IRAK4 (NM_001114182.2). The endogenous control selected was GAPDH. All samples were assayed in triplicate. Thermal cycling conditions were as follows: 2 minutes at 50°C, 10 minutes at 95°C, and, for 40 cycles, 24 seconds at 95°C and 1 minute at 60°C, using the 7900HT Fast Real-Time PCR System. Relative quantification (RQ) values were calculated using the 2^-ΔΔC^t method.

### 2.6. Multiplex ELISA

The following cytokines, tumour necrosis factor alpha (TNF-*α*), interleukin 1*β* (IL-1*β*), interleukin 6 (IL-6), interleukin 8 (IL-8), interferon gamma (IFN-*γ*), interleukin 18 (IL-18), vascular endothelial growth factor (VEGF), erythropoietin (Epo), interleukin 1 receptor antagonist (IL-1ra), and interleukin 10 (IL-10), were analysed using a custom-made MSD® MULTI-SPOT assay plate from Mesoscale (MSD Diagnostics, USA). Extracted peripheral blood serum, as described above, was transferred to a 96-well MSD plate, and these cytokines were assessed as per the manufacturer's instructions. The plate was then analysed on the SECTOR Imager and validated (Meso Scale Discovery, Rockville, MD, USA; http://www.meso-scale.com/). The limits of detection for the individual assays were within expected ranges.

### 2.7. Statistics

Statistical analysis was done using paired and unpaired *t*-tests to compare mean results between two independent cohorts. Two-way ANOVA with Bonferroni multiple comparison tests was also performed to compare responses to different treatments in the two cohorts of interest.

## 3. Results

### 3.1. Monocyte Subset Analysis

Regarding enumeration of monocyte subsets, although children with DS had a higher percentage of intermediate (DS vs. control: 5.2% vs. 4.3%; *p* = 0.16) and nonclassical (DS vs. control: 18.3% vs. 11.5%; *p* = 0.06) subpopulations than controls, there were no statistically significant differences between them. The percentage of classical monocytes was lower in children with DS compared to controls (DS VS. control: 76.4% vs. 84.2%; *p* = 0.048).

### 3.2. TLR2 Expression

**Figure 1:**
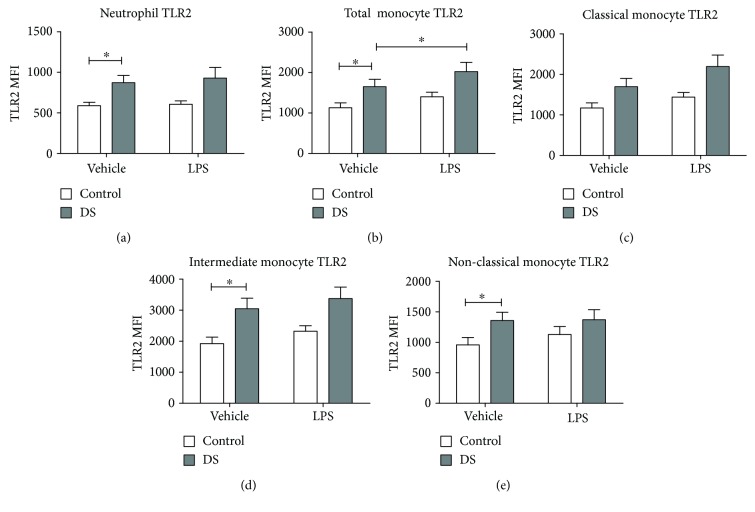
Neutrophil and monocyte Toll-like receptor (TLR2) expression in response to lipopolysaccharide (LPS) in children with Down syndrome (DS, *n* = 20) and controls (*n* = 15). Values expressed as mean channel fluorescence (MFI): (a) neutrophil TLR2; (b) total monocyte TLR2; (c) classical monocyte TLR2; (d) intermediate monocyte TLR2; (e) nonclassical monocyte TLR2. ^∗^*p* < 0.05.

**Figure 2:**
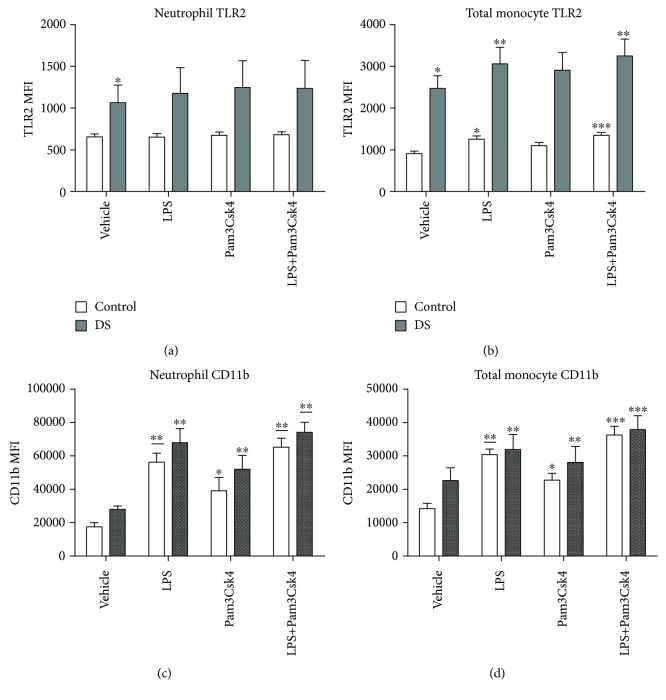
Neutrophil and total monocyte Toll-like receptor (TLR2) and CD11b expression in response to LPS and Pam3Csk4 in children with Down syndrome (DS, *n* = 7) and controls (*n* = 11). Values expressed as mean channel fluorescence (MFI): (a) neutrophil TLR2; (b) total monocyte TLR2 (^∗^*p* < 0.05 vs. vehicle control, ^∗∗^*p* < 0.05 vs. vehicle in the respective cohort, and ^∗∗∗^*p* < 0.05 vs. vehicle, LPS, and Pam3Csk4 in the respective cohort); (c) neutrophil CD11b (^∗^*p* < 0.05 vs. vehicle control, ^∗∗^*p* < 0.05 vs. vehicle in their respective cohort, and ${}^{\underset{¯}{**}}p<0.05$ vs. vehicle and Pam3Csk4 in the respective cohort); (d) total monocyte CD11b (^∗^*p* < 0.05 vs. vehicle control, ^∗∗^*p* < 0.05 vs. vehicle in their respective cohort, ${}^{\underset{¯}{**}}p<0.05$ vs. vehicle and Pam3Csk4 in the respective cohort, and ^∗∗∗^*p* < 0.05 vs. vehicle, LPS, and Pam3Csk4 in the respective cohort).

Neutrophil TLR2 expression was higher in children with DS compared with controls at baseline (*p* = 0.02). After LPS incubation, there was no change in TLR2 expression in either cohort (DS (*p* = 0.25) vs. control (*p* = 0.75); Figure 1(a)). TLR2 expression on total monocytes was also raised at baseline in children with DS versus controls (*p* = 0.04). TLR2 expression post-LPS treatment increased in the DS cohort but not in controls (DS, *p* = 0.0002; control, *p* = 0.12; Figure 2(b)).

Monocyte subset analysis of TLR2 revealed greater expression on intermediate (*p* = 0.01) and nonclassical monocytes (*p* = 0.048) in children with DS when compared to controls at baseline. Classical monocyte TLR2 was higher in children with DS but did not quite reach significance (*p* = 0.06) (Figures 1(c)–1(e)). There were no rises in TLR2 expression in either cohort post-LPS treatment. Intermediate monocytes had the largest mean TLR2 MFI at baseline of any monocyte subpopulation in both children with DS and the control group (DS vs. DS; control vs. control; intermediate vs. classical (*p* = 0.001; *p* = 0.005). Nonclassical monocytes displayed the lowest mean TLR2 at baseline of the three monocyte subsets which was lower than intermediate monocyte TLR2 in both cohorts (Figure 1).

### 3.3. Effect of Pam3Csk4 and LPS on TLR2 Expression

After incubation with Pam3Csk4 and Pam3Csk4 and LPS in combination, there were no rises in TLR2 expression on neutrophils in either cohort (DS (*p* = 0.64) vs. control (*p* = 0.73); Figure 2(a)). TLR2 expression on total monocytes was raised after treatment with LPS and LPS plus Pam3Csk4 in both groups (DS: LPS (*p* = 0.002) and LPS+Pam3Csk4 (*p* = 0.0009); control: LPS (*p* = 0.003) and LPS+Pam3Csk4 (*p* = 0.0001); Figure 2(b)). Treatment with Pam3Csk4 in isolation did not result in any increase in TLR2 in either cohort. The effect of incubating with both Pam3Csk4 and LPS resulted in higher TLR2 expression than did Pam3Csk4 or LPS alone on total monocytes in controls but not in the DS group (vs. Pam3Csk4: DS (*p* = 0.57) and control (*p* = 0.0004); vs. LPS: DS (*p* = 0.14) and control (*p* = 0.002); Figure 2(b)).

For the effect of Pam3Csk4 and LPS on TLR2 expression on monocyte subsets, please see Supplemental Fig. [Supplementary-material supplementary-material-1].

### 3.4. Effect of Pam3Csk4 and LPS on CD11b

There were rises in CD11b after Pam3Csk4, LPS, and LPS plus Pam3Csk4 incubation on neutrophils in both cohorts (Pam3Csk4 (DS, *p* = 0.04; control, *p* = 0.03), LPS (DS, *p* = 0.004; control, *p* ≤ 0.0001), and LPS+Pam3Csk4 (DS, *p* = 0.0004; control, *p* ≤ 0.0001); Figure 2(c)). Treatment with both stimulants resulted in higher CD11b than did that with Pam3Csk4 alone in both groups (DS, *p* = 0.002; control, *p* = 0.0009).

Total monocyte CD11b increased after all treatments in both cohorts (Pam3Csk4 (DS, *p* = 0.03; control, *p* = 0.008), LPS (DS, *p* ≤ 0.0001; control, *p* ≤ 0.0001, and LPS+Pam3Csk4 (DS, *p* = 0.0003; control, *p* ≤ 0.0001); Figure 2(d)), and treatment with both LPS and Pam3Csk4 resulted in higher CD11b expression than did that with either Pam3Csk4 (DS, *p* ≤ 0.0001; control, *p* = 0.0002) or LPS alone (DS, *p* = 0.03; control, *p* = 0.03) also in both groups.

Monocyte subset analysis revealed that intermediate monocyte CD11b was unresponsive to stimulation with LPS and Pam3Csk4 in both cohorts (Supplemental Fig. [Supplementary-material supplementary-material-1]). For the effect of Pam3Csk4 and LPS on CD11b expression on monocyte subsets, please see Supplemental Fig. [Supplementary-material supplementary-material-1].

### 3.5. Effect of SsnB on TLR2 Expression

**Figure 3:**
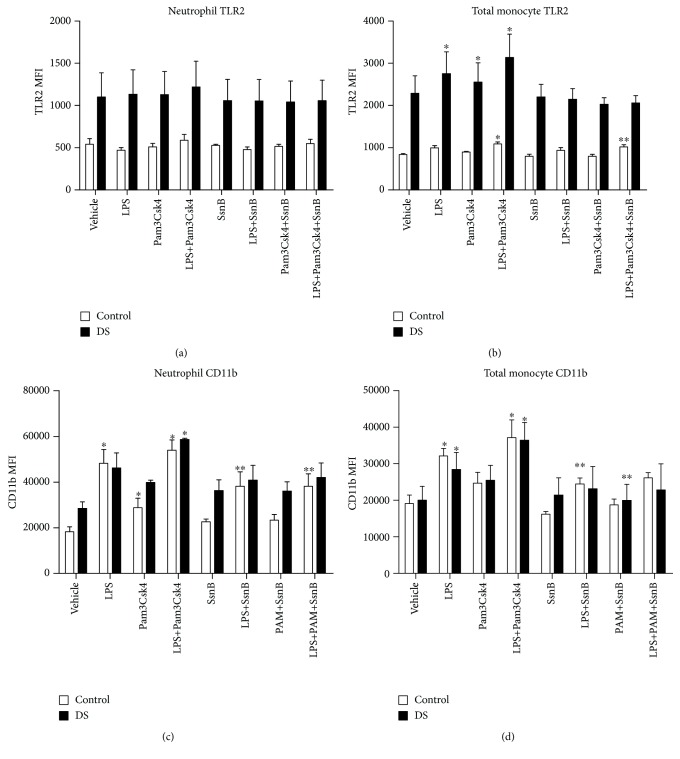
Neutrophil and total monocyte Toll-like receptor 2 (TLR2) and CD11b expression in response to LPS, Pam3Csk4, and SsnB in children with Down syndrome (DS, *n* = 3) and controls (*n* = 3). Values expressed as mean channel fluorescence (MFI): (a) neutrophil TLR2; (b) total monocyte TLR2 (^∗^*p* < 0.05 vs. vehicle in the respective cohort, ^∗∗^*p* < 0.05 vs. LPS in the respective cohort); (c) neutrophil CD11b (^∗^*p* < 0.05 vs. vehicle in the respective cohort, ^∗∗^*p* < 0.05 vs. LPS and LPS+Pam3Csk4 in the respective cohort); (d) total monocyte CD11b (^∗^*p* < 0.05 vs. vehicle in the respective cohort, ^∗∗^*p* < 0.05 vs. LPS and Pam3Csk4 in their respective cohort).

The TLR antagonist SsnB was used to assess its potential as an immunomodulator after stimulation with LPS, Pam3Csk4, and both TLR agonists together. Regarding TLR2 expression on neutrophils, there was no reduction of TLR2 in samples treated with SsnB plus Pam3Csk4 and SsnB plus LPS in children with DS and controls (Pam3Csk4: DS (*p* = 0.076) and control (*p* = 0.78), LPS: DS (*p* = 0.12) and control (*p* = 0.73); Figure 3(a)). In total monocytes, SsnB reduced TLR2 following incubation with LPS plus Pam3Csk4 in controls but not in children with DS (DS, *p* = 0.23; controls, *p* = 0.001; Figure 3(b)). There was also a rise in TLR2 on total monocytes in response to LPS plus Pam3Csk4 in controls. There were no reductions in TLR2 expression following SsnB plus LPS and SsnB plus Pam3Csk4 in both groups (LPS+SsnB: DS (*p* = 0.15) and control (*p* = 0.08), Pam3Csk4+SsnB: DS (*p* = 0.25) and control (*p* = 0.18); Figure 3(b)).

Monocyte subset analysis revealed that SsnB decreased TLR2 on classical and nonclassical monocytes following LPS and LPS plus Pam3Csk4 treatments (classical: LPS (*p* = 0.04) and LPS+Pam3Csk4 (*p* = 0.02); nonclassical: LPS (*p* = 0.03)). Please see Supplemental Fig. [Supplementary-material supplementary-material-1] for the effect of SsnB on TLR2 on monocyte subsets.

### 3.6. Effect of SsnB on CD11b Expression

CD11b expression was reduced on neutrophils following treatment with SsnB plus LPS (*p* = 0.02) and after incubation of LPS with Pam3Csk4 (*p* = 0.004) in the control group; there were also rises in CD11b following treatment with LPS (*p* = 0.05) and LPS and Pam3Csk4 (*p* = 0.02) in this group. In children with DS, there was no change in CD11b on neutrophils following SsnB treatment (LPS+SsnB, *p* = 0.07; LPS+Pam3Csk4, *p* = 0.1; Figure 3(c)). On total monocytes, there was a decrease in CD11b after LPS and SsnB treatment in controls (*p* = 0.01); there was also a rise in CD11b post-LPS incubation (*p* = 0.009). In children with DS, there was a fall in CD11b after SsnB and Pam3Csk4 treatment (*p* = 0.04); in both cohorts, there was no decrease after treatment with SsnB and both proinflammatory stimulants (Figure 3(d)).

Monocyte subset analysis revealed that nonclassical monocyte CD11b was reduced after treatment with LPS, Pam3Csk4, and SsnB in both cohorts (DS, *p* = 0.05; control, *p* = 0.03; Supplemental Fig. [Supplementary-material supplementary-material-1]). Please see Supplemental Fig. [Supplementary-material supplementary-material-1] for the effect of SsnB on CD11b on monocyte subsets.

### 3.7. Gene Expression in Toll-Like Receptor Signalling Pathways

**Figure 4:**
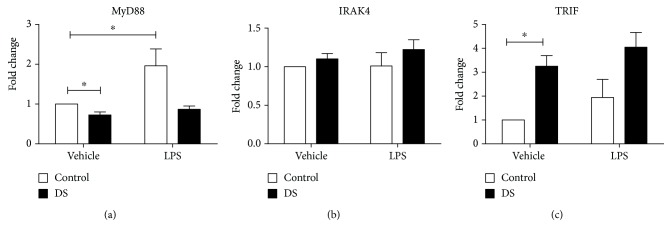
Fold change expression of MyD88 (a), IRAK4 (b), and TRIF (c) in children with DS (*n* = 10) compared to control samples (*n* = 10) at baseline and following treatment with LPS. Statistical significance is ^∗^*p* < 0.05.

Peripheral blood cell expression of MyD88 was lower in children with DS (*p* = 0.001); following LPS treatment, there was a rise in MyD88 in controls (*p* = 0.03), but this was not seen in children with DS (Figure 4(a)). At baseline, TRIF expression was raised in children with DS compared with controls (*p* ≤ 0.0001), and there was no increase in expression following LPS treatment in either group (Figure 4(c)). There were no differences in the expression of IRAK4 between the two groups, and there was an absence of LPS responsiveness in both cohorts (Figure 4(c)).

### 3.8. Immunomodulation and Cytokines: Effects of SsnB—IL-1*β*, TNF-*α*, IL-6, IL-18, VEGF, Epo, IL-8, IFN-*γ*, IL-10, and IL-1ra

**Figure 5:**
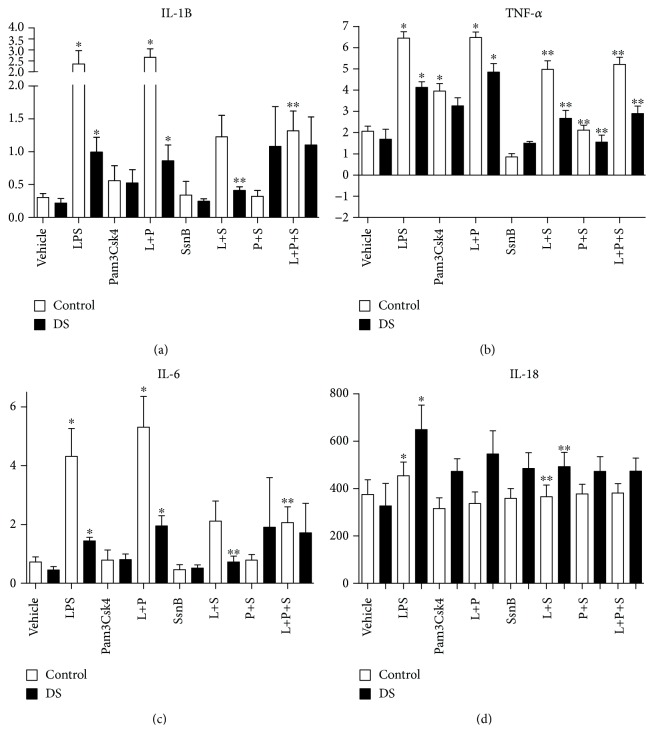
Cytokine levels of IL-1*β*, TNF-*α*, IL-6, and IL-18 in response to LPS (L), Pam3Csk4 (P), and SsnB (S) in children with Down syndrome (DS, *n* = 7) and controls (*n* = 7). Values expressed as pg/mL or in logarithmic scale equivalent. ^∗^*p* < 0.05 vs. vehicle in respective cohort, ^∗∗^*p* < 0.05 vs. respective treatment.

**Figure 6:**
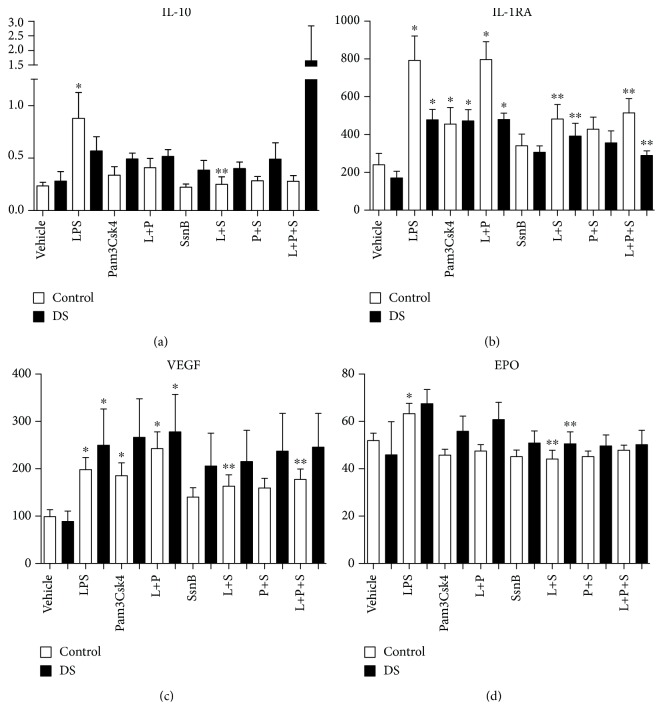
Cytokine levels of IL-10, IL-1RA, VEGF, and EPO in response to LPS (L), Pam3Csk4 (P), and SsnB (S) in children with Down syndrome (DS, *n* = 7) and controls (*n* = 7). Values expressed as pg/mL or in logarithmic scale equivalent. ^∗^*p* < 0.05 vs. vehicle in the respective cohort. ^∗∗^*p* < 0.05 vs. respective treatment.

**Figure 7:**
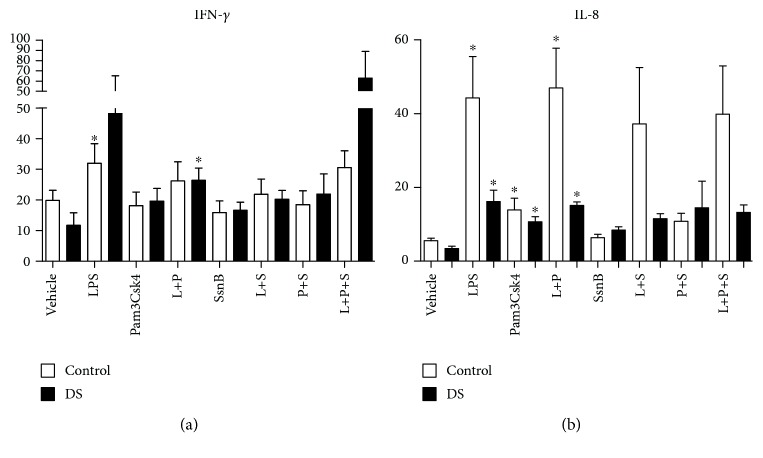
Cytokine levels of IFN-*γ* and IL-8 in response to LPS (L), Pam3Csk4 (P), and SsnB (S) in children with Down syndrome (DS, *n* = 7) and controls (*n* = 7). Values expressed as pg/mL or in logarithmic scale equivalent. ^∗^*p* < 0.05 vs. vehicle in the respective cohort. ^∗∗^*p* < 0.05 vs. respective treatment.

Following stimulation of whole blood with LPS, there were rises in both cohorts of IL-1*β*, TNF-*α*, IL-6, IL-18, IL-8, and VEGF. Significant LPS responsiveness was only seen in control samples for Epo and IFN-*γ* (*p* value < 0.05) (Figures 4–7). The addition of LPS and Pam3Csk4 together also resulted in increases in IL-1*β*, TNF-*α*, IL-6, IL-8, and VEGF in both groups but not in IL-18 or EPO (*p* value < 0.05) and only significantly in the DS cohort for IFN-*γ* (Figures 5–7). There were increases in both cohorts for IL-8, control TNF-*α*, and VEGF after Pam3Csk4 stimulation alone only (*p* value < 0.05), indicating this immunomodulator generates an inferior cytokine response compared with LPS.

When the TLR2 antagonist SsnB was added to LPS, the cytokine production of TNF-*α*, IL-18, and Epo was reduced in both cohorts (*p* value < 0.05) (Figures 5 and 6). IL-1*β* and IL-6 levels fell following SsnB treatment in the DS cohorts only, while VEGF levels were reduced in the control group only. The effects of LPS and Pam3Csk4 together were abrogated by SsnB for IL-1*β*, IL-6, and VEGF in the control group only (*p* value < 0.05). SsnB had the most marked effect on TNF-*α* production, resulting in diminution after its addition to LPS, Pam3Csk, and LPS plus Pam3Csk4 in both cohorts (*p* value < 0.05). SsnB had no effect on IL-8 and IFN-*γ* production (Figures 5–7).

IL-10 release increased following LPS and was reduced by SsnB in controls. Proinflammatory stimulants LPS and Pam3Csk4 resulted in rises in IL-1ra in both cohorts, and this was diminished after the addition of SsnB to LPS and LPS plus Pam3Csk4 in both groups (*p* value < 0.05) (Figure 6).

## 4. Discussion

We have demonstrated increased TLR2 expression on neutrophils, monocytes, and monocyte subsets in children with DS compared to controls. This could be clinically important as excess TLR2 expression is associated with morbidities seen more commonly in DS, such as gram-positive infections [31], autoimmunity [32], and poorer outcomes in sepsis [13]. This is the first time to our knowledge that a dysregulation in TLR2 and its pathways have been described in DS.

We have previously described an increase in TLR4 expression on nonclassical monocytes at baseline and a hyperresponsiveness in neutrophil CD11b following LPS stimulation in children with DS [29]. TLR2 and TLR4 expression is altered in other diseases. In term neonates with late-onset sepsis, TLR4 expression was increased in infants with positive blood cultures and TLR2 expression was raised in those with clinical sepsis [33]. In adult patients with pneumonia, Tang et al. [34] reported an increase in TLR2 and TLR4 expression, as well as raised IL-1, IL-6, and TNF-*α*. Future studies are required to determine the significance of our finding of increased TLR2 expression in DS. Children with DS are at increased risk of RTIs and admission to a hospital with this presentation [18, 35]. Gram-positive bacteria such as *S. pneumoniae* and *S. aureus* are the causative organisms in many of these cases, and TLR2 plays a pivotal role in defence against these pathogens. Studies examining the effects of *S. aureus* and *S. pneumoniae* infection on mice deficient in TLR2 showed a decrease in proinflammatory cytokines and increased mortality [36, 37], highlighting the importance of TLR2 in this clinical context.

Children with DS are also prone to develop recurrent otitis media with effusion (OME) often resulting in conductive hearing loss [38]. *S. pneumoniae* is a significant contributor to this condition and can stimulate persistent low-grade inflammation via TLR2 [39, 40]. Chronic inflammation resulting from persistent infection by many gram-positive and gram-negative bacteria is also seen with increased prevalence in the oral cavity of people with DS [41]. There are studies demonstrating an increase in TLR2 expression in oral mucosal tissue in patients with chronic periodontitis compared with controls [20, 21]. This chronic inflammation and infection secondary to periodontitis may lead to increased numbers of proinflammatory nonclassical monocytes locally and in peripheral circulation [39]; furthermore, a study by Bloemers et al. suggests an increased number of this monocyte subtype in circulation in DS versus controls [40]. We found no significant difference in the cell numbers between the monocyte subpopulations, but the percentage of classical monocytes was significantly less in children with DS compared to controls, as has been described by Bloemers et al. [40]. The differences seen in TLR2 expression between both cohorts could therefore also be influenced by the change in monocyte subpopulations following more frequent bacterial infections in DS. Furthermore, it is difficult to say whether excess gram-positive infection caused greater TLR2 expression or if this receptor is inherently upregulated and or defective from an early age, leading to an increased infectious burden in DS. Overall, persistent inflammation seen in DS could be in part as a result of a dysregulation of TLR2.

In DS, autoimmunity such as arthropathy is also well described [42, 43], with the prevalence being 6 times higher than juvenile idiopathic arthritis in the general population. Increased TLR2 expression on monocyte subsets in patients with active rheumatoid arthritis [44], the mucosa of duodenal samples of patients with coeliac disease (CD) [45, 46], and PBMCs of those with autoimmune thyroid disease have also been reported [47]. Our findings that TLR2 and TLR4 expression and induction are altered in children with DS suggest a possible role for these pathogen receptors in the development of autoimmune diseases in this group.

Taken together, the burden of evidence points to TLR2 as a possible mediator in the pathogenesis of multiple clinical conditions seen more frequently in DS. We have demonstrated that the TLR2/TLR4 antagonist sparstolonin B is effective in reducing TLR2 and CD11b expression in neutrophils and monocytes in children. It has been previously shown that SsnB mediates its anti-inflammatory properties by reducing NF-*κ*B production and attenuating proinflammatory cytokines such as IL-1*β*, IL-6, and TNF-*α* [26]. It appears that SsnB can also abrogate anti-inflammatory cytokine release as it reduces IL-10 and IL-1ra levels in these children. CD11b is a key surface marker upregulated during activation and migration of innate immune cells [48], and it also serves to modulate TLR signalling via both MyD88-dependent and MyD88-independent pathways [49]. We have shown some significant decreases in CD11b expression with SsnB, and this to our knowledge is the first experiment outlining the effects of SsnB on CD11b.

The TLR receptors propagate signals through two main pathways dependent on the utilisation (MyD88 dependent) or not (MyD88 independent) of MyD88. The former is employed by all TLRs except TLR3. The MyD88-dependent pathway results in NF-*κ*B and TNF production. The MyD8-independent pathway signals via TRIF inducing IRF3 and finally leading to IFN-*β* being produced [50]. MyD88 deficiency has been shown to have various clinical consequences: decreased response to IL-1 and IL-18, increased susceptibility to infectious pathogens, and resistance to LPS endotoxin [10, 51, 52]. MyD88 has been shown to be the key in mounting an appropriate immune response to bacterial pneumoniae and streptococci [53]. The deficiency of MyD88 in DS described in our experiment could represent a possible reason for the increased prevalence of infections and poorer clinical outcomes.

While MyD88 expression is decreased in children with DS, we found that TRIF expression is increased. Signalling via the TRIF-dependent pathway leads to a number of important immune responses to infection, enhancing type 1 interferon production and mediating apoptosis, caspase activation, and necroptosis, ultimately leading to a reduction in viral replication and increased bacterial clearance [54]. Children with DS are more susceptible to RTIs, most of viral aetiology, and a paper appraising viral sepsis in children has reported increased expression of TRIF in those affected by this clinical condition [55]. Indeed, TRIF increases the production of important antiviral mediators such as IRF3 and type 1 interferons [56]. However, research in murine models of viral respiratory infection found that excess TRIF signalling had deleterious effects on the host due to excess inflammation, neutrophil recruitment, lung inflammation, and pulmonary oedema [54].

During our experiments, we observed that for some cell populations, positive stimulation did not work for both cohorts, for example, nonclassical monocyte TLR2 and intermediate monocyte CD11b. We previously report similar hyporesponsiveness to LPS in TLR4 expression on nonclassical and intermediate monocytes and CD11b on nonclassical monocytes in children with DS and controls [29]. The reasons for this are unclear as other populations respond significantly, and it could be that receptors on these subsets are inherently hyporesponsive or a degree of endotoxin tolerance may be present; resistance to LPS stimulation caused a blunting of the cellular proinflammatory response [57]. There is some evidence that LPS may act to decrease TLR4 expression on monocyte subsets at 2 h instead of upregulating this receptor. However, there may be a temporal component, as this study points out that TLR2 expression at 20 h on classical monocytes was increased 20-fold, whereas this did not occur for CD16+ subpopulations [58].

## 5. Conclusion

In conclusion, we describe a dysregulation of TLR pathways in DS. There is greater expression of TLR2 on the surface of neutrophils and monocytes. Downstream signalling is altered with reduced MyD88 and increased expression of TRIF, which may represent compensatory upregulation of MyD88-independent pathways. This altered innate immunity may contribute to an increased burden of infections and chronic inflammation in DS. SsnB attenuates proinflammatory mediators and could be of potential therapeutic clinical benefit.

## Data Availability

The data used to support the findings of this study are available from the corresponding author upon request.

## Supplementary Materials

Supplementary Materials Supplemental Figure 1: neutrophil and monocyte Toll-like receptor (TLR2) expression in response to LPS and Pam3Csk4 in children with Down syndrome (DS, *n* = 7) and controls (*n* = 11). Values expressed as mean channel fluorescence (MFI). (a) Neutrophil TLR2, (b) total monocyte TLR2 (^∗^*p* < 0.05 vs. vehicle control, ^∗∗^*p* < 0.05 vs. vehicle in their respective cohort, and ^∗∗^*p* < 0.05 vs. vehicle and Pam3Csk4 in the respective cohort), (c) classical monocyte TLR2 (^∗^*p* < 0.05 vs. vehicle control, ^∗∗^*p* < 0.05 vs. vehicle in their respective cohort, ^∗∗^*p* < 0.05 vs. vehicle and Pam3Csk4 in the respective cohort, and^∗∗∗^*p* < 0.05 vs. vehicle, LPS, and Pam3Csk4 in the respective cohort), (d) intermediate monocyte TLR2 (^∗^*p* < 0.05 vs. vehicle control, ^∗∗^*p* < 0.05 vs. vehicle in their respective cohort, and ^∗∗^*p* < 0.05 vs. vehicle and Pam3Csk4 in the respective cohort), and (e) nonclassical monocyte TLR2 (^∗^*p* < 0.05 vs. vehicle control, ^∗∗^*p* < 0.05 vs. vehicle and LPS in the respective cohort). Supplemental Figure 2: neutrophil and monocyte CD11b expression in response to LPS and Pam3Csk4 in children with Down syndrome (DS, *n* = 7) and controls (*n* = 11). Values expressed as mean channel fluorescence (MFI). (a) Neutrophil CD11b (^∗^*p* < 0.05 vs. vehicle control, ^∗∗^*p* < 0.05 vs. vehicle in their respective cohort, and ^∗∗^*p* < 0.05 vs. vehicle and Pam3Csk4 in the respective cohort), (b) total monocyte CD11b (^∗^*p* < 0.05 vs. vehicle control, ^∗∗^*p* < 0.05 vs. vehicle in their respective cohort, ^∗∗^*p* < 0.05 vs. vehicle and Pam3Csk4 in the respective cohort, and ^∗∗∗^*p* < 0.05 vs. vehicle, LPS, and Pam3Csk4 in the respective cohort), (c) classical monocyte CD11b (^∗^*p* < 0.05 vs. vehicle control, ^∗∗^*p* < 0.05 vs. vehicle in their respective cohort, and ^∗∗^*p* < 0.05 vs. vehicle and Pam3Csk4 in the respective cohort), (d) intermediate monocyte CD11b, and (e) nonclassical monocyte CD11b (^∗^*p* < 0.05 vs. vehicle control, ^∗∗^*p* < 0.05 vs. vehicle in their respective cohort, and ^∗∗^*p* < 0.05 vs. vehicle and LPS in the respective cohort. Supplemental Figure 3: neutrophil and monocyte Toll-like receptor (TLR2) expression in response to LPS, Pam3Csk4, and SsnB in children with Down syndrome (DS, *n* = 3) and controls (*n* = 3). Values expressed as mean channel fluorescence (MFI). (a) Neutrophil TLR2, (b) total monocyte TLR2 (^∗^*p* < 0.05 vs. vehicle in the respective cohort, ^∗∗^*p* < 0.05 vs. LPS+Pam3Csk4 in the respective cohort), (c) classical monocyte TLR2 (^∗^*p* < 0.05 vs. vehicle in the respective cohort, ^∗∗^*p* < 0.05 vs. LPS and LPS+Pam3Csk4 in the respective cohort), (d) intermediate monocyte TLR2, and (e) nonclassical monocyte TLR2 (^∗∗^*p* < 0.05 vs. LPS in their respective cohort). Supplemental Figure 4: neutrophil and monocyte CD11b expression in response to LPS, Pam3Csk4, and SsnB in children with Down syndrome (DS, *n* = 3) and controls (*n* = 3). Values expressed as mean channel fluorescence (MFI). (a) Neutrophil CD11b (^∗^*p* < 0.05 vs. vehicle in the respective cohort, ^∗∗^*p* < 0.05 vs. LPS and LPS+Pam3Csk4 in their respective cohort), (b) total monocyte CD11b (^∗^*p* < 0.05 vs. vehicle in the respective cohort, ^∗∗^*p* < 0.05 vs. LPS and Pam3Csk4 in their respective cohort), (c) classical monocyte CD11b (^∗^*p* < 0.05 vs. vehicle in the respective cohort), (d) intermediate monocyte TLR2, and (e) nonclassical monocyte TLR2 (^∗^*p* < 0.05 vs. vehicle in the respective cohort, ^∗∗^*p* < 0.05 vs. LPS+Pam3Csk4 in the respective cohort).
