# Supplementary material for: Altered Toll-Like Receptor Signalling in Children with Down Syndrome
Source: Mediators Inflamm. 2019 Sep 12;2019:4068734. doi: 10.1155/2019/4068734 (PMC6757445; doi:10.1155/2019/4068734)
Supplement: Supplementary Materials — Supplemental Figure 1: neutrophil and monocyte Toll-like receptor (TLR2) expression in response to LPS and Pam3Csk4 in children with Down syndrome (DS, n = 7) and controls (n = 11). Values expressed as mean channel fluorescence (MFI). (a) Neutrophil TLR2, (b) total monocyte TLR2 (∗p < 0.05 vs. vehicle control, ∗∗p < 0.05 vs. vehicle in their respective cohort, and ∗∗p < 0.05 vs. vehicle and Pam3Csk4 in the respective cohort), (c) classical monocyte TLR2 (∗p < 0.05 vs. vehicle control, ∗∗p < 0.05 vs. vehicle in their respective cohort, ∗∗p < 0.05 vs. vehicle and Pam3Csk4 in the respective cohort, and∗∗∗p < 0.05 vs. vehicle, LPS, and Pam3Csk4 in the respective cohort), (d) intermediate monocyte TLR2 (∗p < 0.05 vs. vehicle control, ∗∗p < 0.05 vs. vehicle in their respective cohort, and ∗∗p < 0.05 vs. vehicle and Pam3Csk4 in the respective cohort), and (e) nonclassical monocyte TLR2 (∗p < 0.05 vs. vehicle control, ∗∗p < 0.05 vs. vehicle and LPS in the respective cohort). Supplemental Figure 2: neutrophil and monocyte CD11b expression in response to LPS and Pam3Csk4 in children with Down syndrome (DS, n = 7) and controls (n = 11). Values expressed as mean channel fluorescence (MFI). (a) Neutrophil CD11b (∗p < 0.05 vs. vehicle control, ∗∗p < 0.05 vs. vehicle in their respective cohort, and ∗∗p < 0.05 vs. vehicle and Pam3Csk4 in the respective cohort), (b) total monocyte CD11b (∗p < 0.05 vs. vehicle control, ∗∗p < 0.05 vs. vehicle in their respective cohort, ∗∗p < 0.05 vs. vehicle and Pam3Csk4 in the respective cohort, and ∗∗∗p < 0.05 vs. vehicle, LPS, and Pam3Csk4 in the respective cohort), (c) classical monocyte CD11b (∗p < 0.05 vs. vehicle control, ∗∗p < 0.05 vs. vehicle in their respective cohort, and ∗∗p < 0.05 vs. vehicle and Pam3Csk4 in the respective cohort), (d) intermediate monocyte CD11b, and (e) nonclassical monocyte CD11b (∗p < 0.05 vs. vehicle control, ∗∗p < 0.05 vs. vehicle in their respective cohort, and ∗∗p < 0.05 vs. vehicle and LPS in the respective coho [file 4068734.f1.docx]

**Supplementary material**

1. **The effect of Pam3Csk4 and LPS on TLR2 expression**
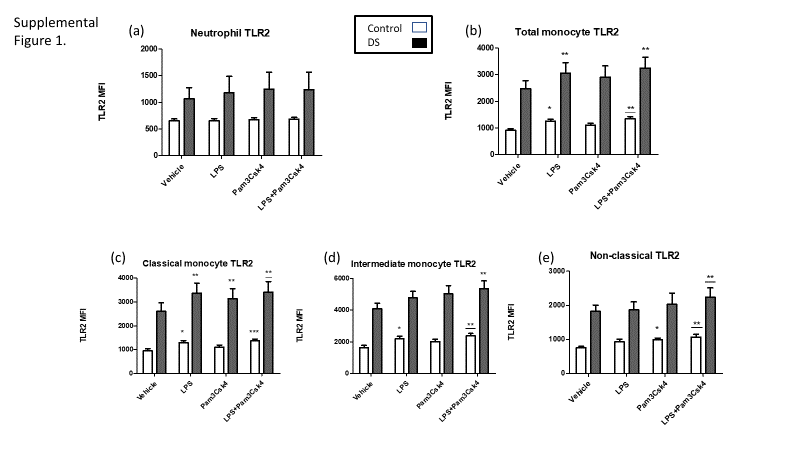


**Supplemental Figure 1: Neutrophil and monocyte Toll-like receptor (TLR2) expression in response to LPS and Pam3Csk4 in children with Down syndrome (DS n=7) and controls (n=11).** Values expressed as mean channel fluorescence (MFI). (a) Neutrophil TLR2; (b) Total monocyte TLR2 *p < 0.05 vs vehicle control; **p <0.05 vs vehicle in their respective cohort; ** p <0.05 vs vehicle and Pam3Csk4 in respective cohort; (c) Classical monocyte TLR2 *p < 0.05 vs vehicle control; **p <0.05 vs vehicle in their respective cohort; ** p <0.05 vs vehicle and Pam3Csk4 in respective cohort; ***p <0.05 vs vehicle, LPS and Pam3Csk4 in respective cohort; (d) Intermediate monocyte TLR2 *p < 0.05 vs vehicle control; **p <0.05 vs vehicle in their respective cohort; ; ** p <0.05 vs vehicle and Pam3Csk4 in respective cohort; (e) Non-classical monocyte TLR2 *p <0.05 vs vehicle control; ** p <0.05 vs vehicle and LPS in respective cohort.

1. **Effect of Pam3Csk4 and LPS on CD11b**

**
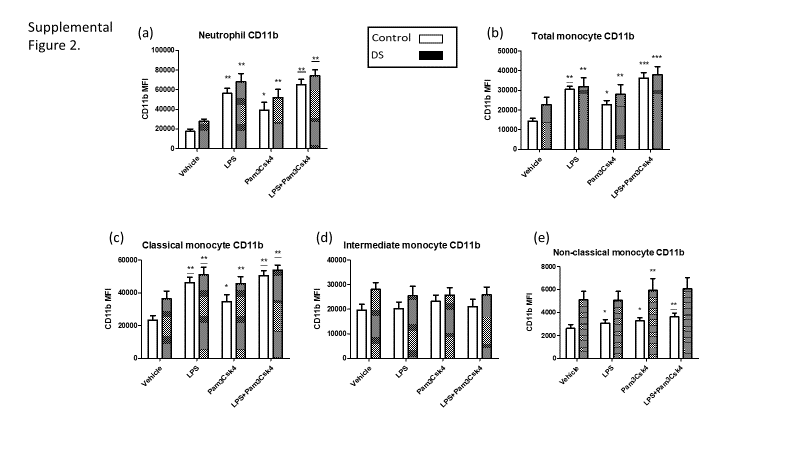
Supplemental Figure 2: Neutrophil and monocyte CD11b expression in response to LPS and Pam3Csk4 in children with Down syndrome (DS n=7) and controls (n=11).** Values expressed as mean channel fluorescence (MFI). (a) Neutrophil CD11b *p < 0.05 vs vehicle control; **p <0.05 vs vehicle in their respective cohort; ** p <0.05 vs vehicle and Pam3Csk4 in respective cohort;; (b) Total monocyte CD11b *p < 0.05 vs vehicle control; **p <0.05 vs vehicle in their respective cohort; ** p <0.05 vs vehicle and Pam3Csk4 in respective cohort; ***p <0.05 vs vehicle, LPS and Pam3Csk4 in respective cohort; (c) Classical monocyte CD11b *p < 0.05 vs vehicle control; **p <0.05 vs vehicle in their respective cohort; ** p <0.05 vs vehicle and Pam3Csk4 in respective cohort; (d) Intermediate monocyte CD11b; (e) Non-classical monocyte CD11b *p <0.05 vs vehicle control; **p <0.05 vs vehicle in their respective cohort; ** p <0.05 vs vehicle and LPS in respective cohort.

1. **Effect of SsnB on TLR2 expression**


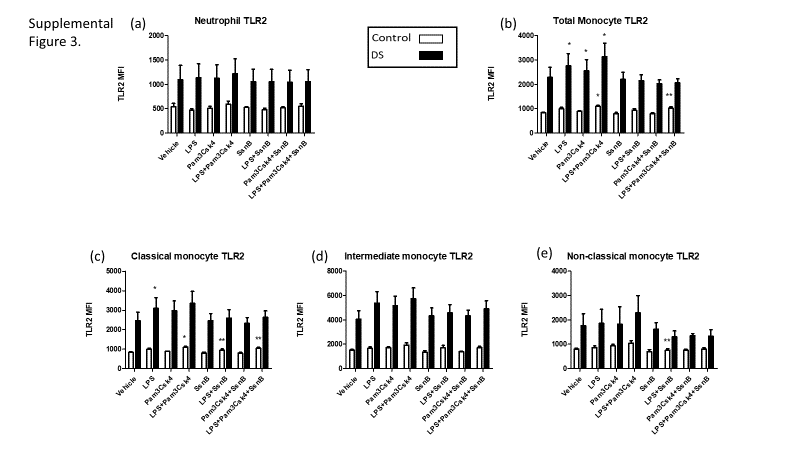


**Supplemental Figure 3: Neutrophil and monocyte Toll-like receptor (TLR2) expression in response to LPS, Pam3Csk4 and SsnB in children with Down syndrome (DS n=3) and controls (n=3).** Values expressed as mean channel fluorescence (MFI). (a) Neutrophil TLR2; (b) Total monocyte TLR2 *p < 0.05 vs vehicle in respective cohort; **p <0.05 vs LPS+Pam3Csk4 in respective cohort; (c) Classical monocyte TLR2 *p < 0.05 vs vehicle in respective cohort; **p <0.05 vs LPS and LPS+Pam3Csk4 in respective cohort; (d) Intermediate monocyte TLR2; (e) Non-classical monocyte TLR2 **p <0.05 vs LPS in their respective cohort.

1. **Effect of SsnB on CD11b expression**


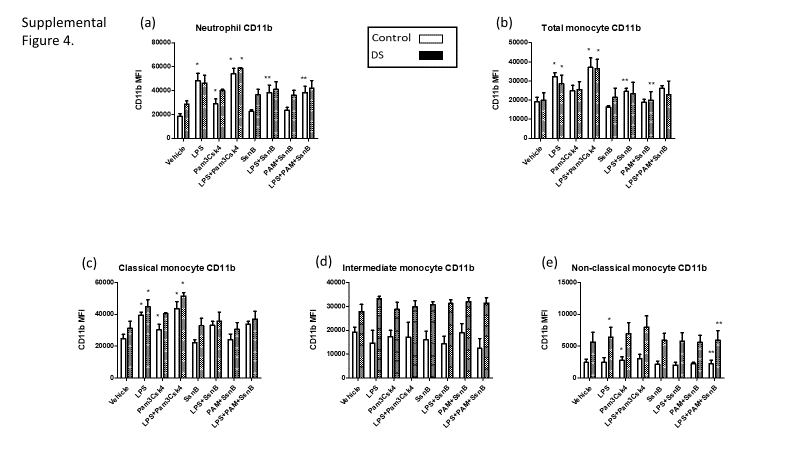


**Supplemental Figure 4: Neutrophil and monocyte CD11b expression in response to LPS, Pam3Csk4 and SsnB in children with Down syndrome (DS n=3) and controls (n=3).** Values expressed as mean channel fluorescence (MFI). (a) Neutrophil CD11b *p < 0.05 vs vehicle in respective cohort; **p <0.05 vs LPS and LPS+Pam3Csk4 in their respective cohort; (b) Total monocyte CD11b *p < 0.05 vs vehicle in respective cohort; **p <0.05 vs LPS and Pam3Csk4 in their respective cohort; (c) Classical monocyte CD11b *p < 0.05 vs vehicle in respective cohort; (d) Intermediate monocyte TLR2; (e) Non-classical monocyte TLR2 *p < 0.05 vs vehicle in respective cohort; **p <0.05 vs LPS+Pam3Csk4 in respective cohort.
